# Impact of Radiotherapy on Malfunctions and Battery Life of Cardiac Implantable Electronic Devices in Cancer Patients

**DOI:** 10.3390/cancers15194830

**Published:** 2023-10-02

**Authors:** Dominik Lisowski, Paul Lutyj, Arya Abazari, Stefan Weick, Jan Traub, Bülent Polat, Michael Flentje, Johannes Kraft

**Affiliations:** 1 Department of Radiation Oncology, University Hospital Würzburg, 97080 Würzburg, Germanykraft_j1@ukw.de (J.K.); 2 Department of Internal Medicine I, Division of Cardiology, University Hospital Würzburg, 97080 Würzburg, Germany

**Keywords:** battery depletion, cardiac implantable electronic devices (CIED), cardiac resynchronization therapy (CRT), implantable cardioverter defibrillator (ICD), CIED malfunction, pacemaker (PM), radiotherapy (RT)

## Abstract

**Simple Summary:**

Ionizing radiotherapy (RT) can cause malfunctions to cardiac implantable electronic devices (CIEDs), posing a risk to patient safety. The aim of the study was to quantify and categorize the risk of CIED malfunction during RT and to analyze national guidelines/recommendations for adequate patient safety. We found that the CIED malfunction rate is low (2.8%) in cancer patients receiving RT. The probability of CIED malfunction does not correlate with the applied photon energy, the dose at the CIED nor the treated area. Battery depletion occurs very rarely (1.2%). The national guidelines strongly differ in regard to safety the recommendations for patients with a CIED during and after RT.

**Abstract:**

Purpose: This study analyses a large number of cancer patients with CIEDs for device malfunction and premature battery depletion by device interrogation after each radiotherapy fraction and compares different guidelines in regard to patient safety. Methods: From 2007 to 2022, a cohort of 255 patients was analyzed for CIED malfunctions via immediate device interrogation after every RT fraction. Results: Out of 324 series of radiotherapy treatments, with a total number of 5742 CIED interrogations, nine device malfunctions (2.8%) occurred. Switching into back-up/safety mode and software errors occurred four times each. Once, automatic read-out could not be performed. The median prescribed cumulative dose at planning target volume (PTV) associated with CIED malfunction was 45.0 Gy (IQR 36.0–64.0 Gy), with a median dose per fraction of 2.31 Gy (IQR 2.0–3.0 Gy). The median maximum dose at the CIED at time of malfunction was 0.3 Gy (IQR 0.0–1.3 Gy). No correlation between CIED malfunction and maximum photon energy (*p* = 0.07), maximum dose at the CIED (*p* = 0.59) nor treatment localization (*p* = 0.41) could be detected. After excluding the nine malfunctions, premature battery depletion was only observed three times (1.2%). Depending on the national guidelines, 1–9 CIED malfunctions in this study would have been detected on the day of occurrence and in none of the cases would patient safety have been compromised. Conclusion: Radiation-induced malfunctions of CIEDs and premature battery depletion are rare. If recommendations of national safety guidelines are followed, only a portion of the malfunctions would be detected directly after occurrence. Nevertheless, patient safety would not be compromised.

## 1. Introduction

Since the first implantation in 1958, cardiac implantable electronic devices (CIEDs), which include permanent pacemakers (PMs), implantable cardioverter defibrillators (ICDs) and devices for cardiac resynchronization therapy (CRT), have played a crucial role in potentially life-saving treatments for various cardiac diseases. Currently, a steady increase of implantation rates can be observed with high incidence variations between individual countries [1,2]. Due to an aging population and a higher prevalence of cancer, the number of patients with CIEDs receiving radiotherapy (RT) is also expected to rise [3]. RT can induce malfunctions in CIEDs by an aberrant accumulation of charge or current flow within the irradiated semiconductor materials or by a single-event upset (SEU) induced by photoneutrons, particularly in the random access memory (RAM) [4,5]. The malfunctions of CIEDs are various, ranging from altered stimulation or sensing to the complete loss of function of the device. In addition, the rapid discharge of the battery, resulting in shorter battery life, may rarely occur. Potential errors of CIEDs can lead to life-threatening events, especially in PM-dependent patients. Therefore, patients with CIEDs must be subject to special monitoring during RT. According to a limited number of studies with mostly small study groups, the incidence rates for malfunctions strongly vary, ranging between 0% and 25% [6]. There is a lack of large-scale studies with close monitoring of CIEDs to accurately detect not only permanent but also transient malfunctions. Because of this paucity of data, many recommendations of national guidelines are only based on expert opinions [4,5,6,7,8,9,10,11]. To obtain a proper level of monitoring for CIEDs, the recommendations classify patient groups by their risk probability and the severity of CIED malfunction [4,5]. Yet, definitions of the patient groups and recommendations for each group strongly differ between the national safety guidelines. In this single-centered, retrospective study, we analyzed a large number of patients with CIEDs for device malfunction and premature battery depletion by device interrogation after each radiotherapy fraction.

## 2. Materials and Methods

All cancer patients who received RT at our institution from 2007 to 2022 and had CIED interrogations before the first RT treatment and after every radiotherapy fraction were included. For patients with multiple radiotherapy treatment series, each individual series was separately analyzed. Patients receiving brachytherapy only were excluded. The primary endpoint of our study was CIED malfunction. The secondary endpoints were battery discharge during RT, calculated and actual battery life, as well as CIED replacement. The CIED data readout was examined for malfunction, battery status, and replacement. CIED malfunction was defined as any abnormality compared with the initial setup before RT. Malfunctions included, but were not limited to, electrical reset to backup mode or other software errors, loss of telemetry, unplanned changes in lead parameters, noise oversense with or without symptomatic pacing inhibition or shock therapy. Battery discharge was defined as a decrease in battery voltage, a decrease of battery power displayed in percentage, a decrease in magnetic frequency or an increase in impedance by more than 10% between the initiation and end of RT. Different safety guidelines for RT in patients with CIEDs were evaluated for proper device failure detection and patient safety using the actual courses of the detected CIED malfunctions of this study [4,5,6,7,8,9,11]. 

After computed tomography (CT) acquisition, planning target volume (PTV) was delineated and radiotherapy treatment was planned using Pinnacle^3^ (Philips Radiation Oncology Systems, Fitchburg, WI, USA) or Eclipse treatment planning software version 15.6 (Varian Medical Systems, Palo Alto, CA, USA). If the CIED generator was visible on simulation CT, the device was delineated and considered as an organ at risk (OAR). Leads of CIEDs were not delineated. Dose at the CIED was minimized in the best possible way without compromising the dose in PTV. After treatment planning, the estimated radiation dose was calculated for the CIED. In case the CIED was not depicted on the simulation CT, no delivered radiation dose on the CIED was assumed. The techniques used for treatment included 3D conformal radiotherapy (3D-CRT), intensity modulated radiotherapy (IMRT), and volumetric intensity modulated arc therapy (VMAT). For conventional radiotherapy, external beams were directly adjusted without CT and, if applicable, positioning was confirmed using digitally reconstructed radiography (DRR) and portal images. IMRT was delivered as a step-and-shoot technique with 3–9 fields. VMAT consisted of one to four dynamic arcs. The use of flattening filter-free radiotherapy (FFF-RT) was also permitted for treatment of patients with CIEDs. RT was conducted using a Siemens PRIMUS (Siemens Healthcare GmbH, Erlangen, Germany), ELEKTA Synergy (Elekta AB, Stockholm, Sweden) or Varian Halcyon and Ethos (Varian Medical Systems, Palo Alto, CA, USA) linear accelerators (LINACs). During treatment, an external defibrillator was always available outside the treatment rooms and the assigned emergency team was able to reach the patient within 5 min in case of emergency. Tachyarrhythmia detection and therapy of ICDs were always suspended by placement of a magnet over the ICD during RT sessions.

All statistical analyses were performed with SPSS Statistics 29.0 (IBM, Armonk, NY, USA) and graphically illustrated using GraphPad Prism version 5 (GraphPad Software, San Diego, CA, USA). The threshold for statistical significance was set at a two-sided *p* < 0.05. Variables are expressed as mean ± SD if not otherwise stated. To determine the independence or association between categorical variables, the chi-squared test was performed. The binary logistic regression analysis was applied to examine the relationship between continuous and dichotomous variables.

## 3. Results

### 3.1. Results on Patient and Treatment Characteristics

**Figure 1:**
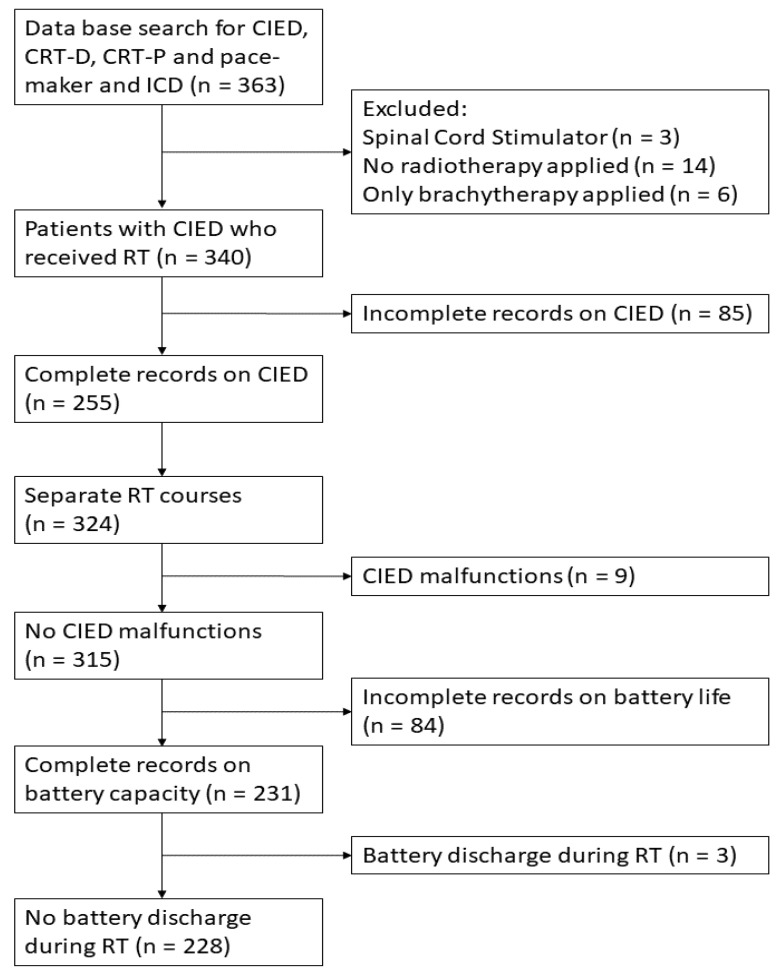
Flowchart of the study population. Abbreviations: CIED = cardiac implantable electronic device; CRT-D/CRT-P = cardiac resynchronization therapy with defibrillator/pacemaker; ICD = implantable cardioverter defibrillator; PM = pacemaker; RT = radiotherapy.

Among the 340 cancer patients with a CIED, 255 patients, receiving 324 series of RT with a total of 5742 interrogations, were included and subsequently analyzed (Figure 1). Out of the 255 patients, 55 patients had more than one series of RT. Median age at time of the first included radiation series was 75.3 years (IQR 68.8–80.8) and 178 patients were men (69.8%). The median Karnofsky performance status at the initiation of RT was 80 points, ranging from 40 to 100 points. Out of the 255 CIEDs, 171 pacemakers, 68 ICDs, and 16 CRT devices were evaluated. Devices from the manufacturers Biotronik, Medtronic and St. Jude Medical, currently Abbott, were the most frequently implanted CIEDs in our cohort with 150, 42 and 23 devices, respectively. The median age of the CIED at initiation of RT was 3.3 years (IQR 1.4–5.8). Atrioventricular block, tachyarrhythmias and conditions associated with a reduced ejection fraction were the most common reasons for the implantation of a CIED. DDD(R) and VVI(R) were the most common pacing modes, with 104 and 51 cases, respectively. All patient and device characteristics are summarized in Supplementary Table S1. 

The most common localizations for RT were the thorax, pelvis and head/neck, with 119, 89 and 73 treatments, respectively. The median total dose delivered to the patient was 47.5 Gy (IQR 30.0–60.4 Gy). The median dose per fraction was 2.67 Gy (IQR 2.1–4.0 Gy). The calculated median dose at the CIED was 0.9 Gy (IQR 0.3–2.0 Gy), with a median maximum dose of 2.4 Gy (0.6–6.0 Gy). The mean PTV was 714.8 cm^3^ (± 662.7 cm^3^), and the mean boost PTV was 220.1 cm^3^ (± 246.4 cm^3^). Boost radiotherapy was performed in 138 radiotherapy treatments (42.6%). Photon beams with less than 10 MV energy were used in 202 treatments (62.3%), whereas 111 treatments (34.3%) had at least one photon beam with equal or more than 10 MV energy. Out of 324 radiotherapy treatments, eleven (3.4%) consisted of treatments with electron beams only. 3D-CRT and VMAT were the most common radiation techniques, with 140 (35.8%) and 121 (31.0%) applications, respectively. FFF-RT was only performed in 24 cases (6.1%). Different radiation techniques could be performed in the same radiotherapy session. All treatment characteristics are summarized in Table 1. 

### 3.2. CIED Malfunctions

Among 324 RT treatments with complete data on the CIED, nine device malfunctions (2.8%) occurred, whereby one person simultaneously received two radiotherapy treatments of different bone metastases in close proximity when the CIED malfunction occurred (Table 2). As it is not distinguishable which radiotherapy treatment caused the malfunction in this patient, we considered both treatments as contributing to the malfunction of the CIED equally. The median cumulative radiation dose at PTV that was associated with CIED malfunction was 45.0 Gy (IQR 36.0–64.0 Gy), with a median dose per fraction of 2.31 Gy (IQR 2.0–3.0 Gy). The median maximum dose at CIED calculated in one voxel at time of malfunction was 0.3 Gy (IQR 0.0–1.3 Gy). Malfunction occurred in four ICDs, three CRT devices and two PMs. The median time between CIED implantation and malfunction was 34 months (range 4–78 months). In six cases with CIED malfunctions (66.7%), treatment with photon energies ≥ 10 MV was conducted. No CIED malfunctions were observed during RT with electron beams. Six cases of malfunction (66.7%) occurred during RT of structures in the thorax, two during RT of the pelvis (22.2%) and one in the abdomen region (11.1%). No correlation between CIED malfunction and maximum photon energy (*p* = 0.07), maximum dose at the CIED (*p* = 0.59) nor treatment localization (*p* = 0.41) could be detected. The two most common malfunctions were the switch of the CIED into back-up/safety mode (four times) and software errors with deactivation of functions or mode switches (four times). In one case, the automatic read-out could not be performed. One device in safety mode could not be reinitiated and had to be exchanged. In the other eight cases, the CIEDs could be reprogrammed in the clinic. All information about device malfunctions is summarized in Table 2. In accordance with the German Society for Radiation Oncology (DEGRO)/German Cardiac Society (DGK) guideline, the Dutch practical guideline and the Polish expert opinion, nine, five and four malfunctions would have been detected on the day of occurrence, respectively [5,9,11]. Following the 2017 Heart Rhythm Society (HRS) expert consensus, the American Association of Physicists in Medicine (AAPM) TG-34 guideline or the Italian or French consensus would have only resulted in the detection of one out of nine malfunctions on the day of occurrence [4,6,7,8]. However, patient safety would not have been compromised in any of our cases.

### 3.3. Premature Battery Depletion

**Figure 2:**
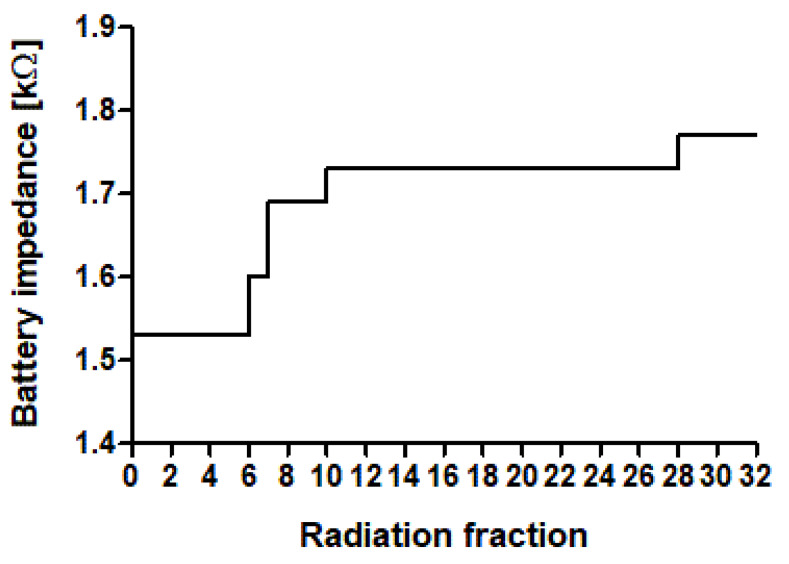
Time course of battery discharge by increase of battery impedance over the treatment period. The patient received a total of 32 sessions of radiotherapy. The session number is plotted on the *X*-axis. The battery impedance in kΩ is plotted on the *Y*-axis.

Out of 315 RT treatments without malfunction, complete battery records were available in 231 cases (Figure 1). A distinctive decrease in battery capacity was only observed three times (1.2%). Battery capacity loss was not gradual but consisted rather of single decreases that accumulated over the period of RT. The increase in battery impedance recorded over the entire treatment period of one case is exemplarily displayed in Figure 2. A recovery in battery capacity after a decrease was never observed. In all three cases, battery capacity decline did not reach the elective replacement indication (ERI). All three devices with battery discharge were pacemakers, two devices were from the manufacturer Biotronik and one from Sorin. No correlation between battery discharge and maximum photon energy (*p* = 0.49), maximum dose at the CIED (*p* = 0.06) nor treatment localization (*p* = 0.88) could be detected.

## 4. Discussion

To the best of our knowledge, this study represents the largest cohort of patients who have undergone CIED interrogation after every fraction of RT. Such a close monitoring of a cohort was only possible due to the strict recommendations of the DEGRO/DKG guidelines, which recommends a CIED interrogation after each fraction of RT independently of the risk stratification [5]. The detected malfunction rate of 2.8% in our cohort is relatively low compared with the malfunction rates in previously published reports, although rates strongly differ when compared [12,13,14,15,16,17,18,19]. For instance, Kappa et al., Ferrara et al. and Levis et al. reported no malfunctions at all in their relatively small cohorts (less than 50 CIEDs) [20,21,22]. In larger cohorts with more than 100 CIEDs, the malfunction rates differ between 0.6 and 7.8% [12,14,15,16,18,19,23,24,25,26,27]. This might be due to different compositions of analyzed cohorts or different timings of interrogation. Cardiological follow-up visits in our institution after RT were rarely performed because patients in our cohort preferably visited the referring cardiologists in other institutes or private practices for further CIED checkups. Hence, a long-term follow-up is missing in this study, though it is questionable whether RT has long-term impacts on CIEDs in addition to immediate malfunctions. Data on the general malfunction rates of CIED generators are scarce. Maisel et al. reported that the annual malfunction replacement rate per 1000 implants was 1.4 for PMs in 2002 and 36.4 for ICDs in 2001 [28]. Despite an intensive database search, more recent data on general CIED generator malfunctions could not be found.

In concordance with the only meta-analysis on CIED malfunctions, we observed relatively more malfunctions in ICDs (4/68) and CRT devices (3/16) than in PMs (2/171) [29]. Furthermore, a significant correlation between malfunction in CIEDs and maximum photon energy was almost reached in our cohort (*p* = 0.07), suggesting that neutron-producing energies might increase the risk of malfunction, as suggested in previous publications [29,30]. It is speculated that this is due to the increased amount of boron-10 in the lower intermetal dielectric layers of the integrated circuit. Boron-10 interacts with the neutrons, resulting in the production of lithium-7 and α-particles in the immediate vicinity of the active circuit [31]. As four CIED malfunctions were detected at treatments with a calculated cumulative dose of 0 Gy at the CIED but with photon energies ≥ 10 MeV, we assume that the scattered neutrons produced by high-energy beams might cause malfunction independently from the distance to the CIED [32]. However, other mechanisms, such as electromagnetic interference (EMI) with scattered radiofrequency (RF) waves emitted by the LINAC due to insufficient shielding, could also be potential causes for CIED malfunction and cannot be completely excluded [33,34]. EMI can lead to various effects on CIEDs, ranging from benign interference that may not affect device function to potentially more serious consequences. As EMI can be interpreted by the PM as an intrinsic cardiac signal, pacing can be inhibited, leading to bradycardia and potentially cardiac arrest [33]. In ICDs, oversensing due to EMI can result in unnecessary shock delivery [33]. In our study, EMI was not detected, mainly because RF emissions of the LINACs were very minor when measured. Another cause of CIED malfunction is the damage of direct ionizing radiation on complementary metal oxide semiconductor (CMOS) devices that are built in all modern CIEDs. CMOS are susceptible to direct radiation due to the buildup of radiation-induced charge in the SiO_2_ layer causing a shift in the threshold voltage and consequently to aberrant behaviors [4].

Our cohort predominantly consisted of patients receiving RT in the region of the thorax, which might consequently lead to higher dose contributions to the CIEDs, although our calculated dose at the CIED was within the range of a previously published report [35]. There was no significant correlation between malfunction and treatment location; however, six out of nine malfunctions occurred in patients who received RT in the thoracic region, suggesting that close proximity of the target volumes to CIEDs increases the probability of malfunctions. Probably due to the general low number of malfunctions, a positive correlation between malfunction and proximity of treatment localization did not reach statistical significance. Modern RT techniques such as IMRT and VMAT enable high-precision radiation, minimizing the dose at OARs and at CIEDs. Nevertheless, even low doses at CIEDs are still problematic and can cause irreversible damage to the devices and consequently to the patient [36]. In order to minimize dose at CIEDs, non-coplanar beams were used in our cohort group whenever feasible to further decrease the doses at the CIEDs.

Besides assessment of CIED malfunctions, this study also focused on the battery life of CIEDs under RT. Hence, we excluded premature battery depletion from the list of malfunctions and analyzed all CIEDs without malfunctions for a decrease in battery capacity separately. We could detect premature battery depletion in three PMs under RT. The decrease in battery capacity was never gradual but consisted of single decreases that accumulated over the period of the RT. Figure 2 graphically depicts the decrease in the battery capacity of a PM by the increase in the battery impedance. Premature battery depletion was already observed in earlier published studies but is generally a very rare occurrence, particularly in PMs [37,38,39]. Batteries based on lithium systems have been installed in CIEDs since the 1970s and have been by far the most frequently used batteries in CIEDs up to now [40]. The exact mechanism of battery depletion during RT is not yet fully understood, but SEUs are believed to play a crucial role in premature battery discharge.

Our study included only patients who received CIED interrogation after every radiotherapy fraction. Different national guidelines set standards for the frequency of the interrogations as well as for the additional safety measures during and after RT. A closer look reveals significant differences between the national recommendations, especially in regard to the frequency of interrogations (Table 3). We simulated the scenarios of our nine device malfunctions in accordance with different guidelines and found out that not all of the errors that occurred would have been detected directly after the radiotherapy session. Nevertheless, patient safety would not have been compromised in any of the cases. Only in the AAPM TG-34 guideline and the in Italian consensus document might the safety precautions for intermediate-risk patients be insufficient, as both lack the recommendation of weekly interrogations for PM-dependent patients, which might pose a threat to patients’ health by delayed recognition of CIED malfunctions [4,6]. A flaw in the German and Dutch guidelines is the missing safety recommendation for handling patients who receive RT with the production of secondary neutrons. Both guidelines strongly recommend using only photon beam energies ≤ 10 MV. For certain situations, however, higher beam energy results in a more conformal dose distribution at PTV. In such cases, the better dose distribution should be carefully considered against the higher risk of CIED malfunction. In addition, the increased use of proton therapy, which produces clinically significant amounts of secondary neutrons, has to be taken into account and might eventually lead to a revision of both guidelines [8]. We classified patients receiving radiotherapy with production of secondary neutrons as high-risk unless otherwise stated in the corresponding guideline.

An adequate risk stratification for patients with a CIED enables necessary safety precautions without excessive resource consumption and overload of the healthcare system. Due to the reported low malfunction rate of CIEDs during RT, interrogations of CIEDs after every RT session represent an excessive safety precaution for low-risk patients and patients with an end-stage disease. Remote monitoring during and after RT can additionally help to monitor patients without time-consuming interrogations at the treatment site [41].

## 5. Conclusions

Malfunctions of CIEDs are rare and premature battery depletion is highly unlikely in real-world cancer patients during RT. If excessive battery loss occurs, it consists of single decreases that accumulate over the time of the RT. Standardized national guidelines are essential to ensure the safety of patients with CIEDs undergoing RT. They greatly differ in their recommendations but do not compromise patient safety.

## Figures and Tables

**Table 1:** Radiation treatment characteristics (n = 324).

Variable		Number	(%)
Localization			
	Head and neck	73	22.5%
	Thorax	119	36.7%
	Abdomen	25	7.7%
	Pelvis	89	27.5%
	Upper extremities	7	2.2%
	Lower extremities	11	3.4%
Radiation therapy dose in Gy			
	Median dose (IQR)	40.05	(30.0–59.3)
	Boost dose (IQR)	10.0	(6.9–14.0)
	Total median dose (IQR)	47.5	(30.0–60.4)
	Total median dose per fraction (IQR)	2.67	(2.1–4.0)
Calculated dose of CIED in Gy (n = 122)			
	Median dose (IQR)	0.9	(0.3–2.0)
	Median maximum dose (IQR)	2.4	(0.6–6.0)
Target volume in cm^3^			
	Mean planning target volume (SD)	714.8	(662.7)
	Mean boost target volume (SD)	220.1	(246.4)
Beam modality			
	Max. photon beam energy of 18 MV	55	17.0%
	Max. photon beam energy of 10 MV	56	17.3%
	Max. photon beam energy of 6 MV	202	62.3%
	Electron therapy only	11	3.4%
Radiation technique (n = 391)			
	3D-CRT	140	35.8%
	IMRT	46	11.8%
	VMAT	121	31.0%
	Radiotherapy without simulation CT	60	15.3%
	FFF-RT	24	6.1%

Abbreviations: 3D-CRT = 3D conformal radiotherapy; CIED = cardiac implantable electronic device; CT = computed tomography; FFF-RT = flattening filter-free radiotherapy; IMRT = intensity modulated radiotherapy; IQR = interquartile range; Max. = maximum; SD = standard deviation; VMAT = volumetric intensity modulated arc therapy.

**Table 2:** Patient and Event Details for Cases of CIED Malfunction.

No.	Device Type	Manufacturer	Model	Anatomic Area Irradiated	Total Dose/Number of Fractions Planned	Dose until Malfunction	RT Sessions Applied until Malfunction	Max. Dose at CIED at Time of Malfunction	Beam Type	Maximum Beam Energy	Description
(1.)	PM	Biotronik	Entovis DR-T	Lung, left upper lobe	45 Gy/18 fx	42.5 Gy	17	2.7 Gy	Photons	10 MV	Device in back-up mode
(2.)	ICD	Biotronik	Lumax DR-T	Mediastinum, interpleural space	9 Gy/3 fx	9 Gy + 15.4 Gy = 24.4 Gy	10	1.25 Gy	Photons	18 MV	Software error with multiple mode switches
					61.6 Gy/28 fx						
(3.)	PM	Biotronik	Cylos DR	Left groin	30 Gy/15 fx	30 Gy	15	0 Gy	Photons	18 MV	Device in safety mode, reprogramming unsuccessful, device exchanged
(4.)	CRT-P	Boston Scientific	Contak Renewal TR	Left breast	50 Gy/25 fx	36 Gy	18	2.1 Gy	Photons	6 MV	Failure of automatic read-out
(5.)	CRT-D	Biotronik	Itrevia 7 HF-T/QP	Lower abdomen	36 Gy/18 fx	(errors several times during RT)	(errors several times during RT)	0 Gy	Photons	18 MV	Functional software errors occurring several times during RT
(6.)	CRT-D	Biotronik	Rivacor 5 HF-T/QP	Prostate	76.23/33 fx	43.89 Gy	19	0 Gy	Photons	10 MV	Device in safety mode
(7.)	ICD	Biotronik	Lumax 340 VR-T	Lower Esophagus	64 Gy/32 fx	26 Gy	13	0 Gy	Photons	18 MV	Device in safety mode
(8.)	ICD	Biotronik	Lumax 740 VR-T DX	Thoracic spine (6–10)	30 Gy/10 fx	15 Gy	5	0.36 Gy	Photons	6 MV	Antitachycardia functions were deactivated
	ICD	Biotronik	Lumax 740 VR-T DX	Left ribs (6–10)	30 Gy/10 fx	15 Gy	5	0.25 Gy	Photons	6 MV	Antitachycardia functions were deactivated

Abbreviations: CIED = cardiac implantable electronic device; CRT-D/P = cardiac resynchronization therapy with defibrillator/pacemaker; ICD = implantable cardioverter defibrillator; PM = pacemaker; RT = radiotherapy.

**Table 3:** Comparison of different guidelines/consensuses/expert opinions.

		DEGRO/DKG Guideline	Dutch Guideline	AIAC/AIRO/AIFM Consensus	SFRO Consensus	PTK/PTRO Opinion	HRS Consensus	AAPM TG-34 Guideline
Risk stratification								
	Low risk	- CIED dose < 2 Gy without pacemaker dependency or history of prior ventricular fibrillation	- CIED dose < 2 Gy without pacemaker dependency	- CIED dose ≤ 2 Gy without pacemaker dependency or frequent ICD intervention	- CIED dose ≤ 5 Gy without pacemaker dependency and without production of secondary neutrons	- PM dose < 5 Gy without pacemaker dependency	- CIED dose ≤ 5 Gy without pacemaker dependency and without production of secondary neutrons	- CIED dose < 2 Gy without pacemaker dependency
	Intermediate risk	- CIED dose 2–10 Gy without pacemaker dependency or history of prior ventricular fibrillation - CIED dose < 2 Gy with pacemaker dependency or history of prior ventricular fibrillation	- CIED dose 2–10 Gy without pacemaker dependency or history of prior ventricular fibrillation - CIED dose < 2 Gy with pacemaker dependency or history of prior ventricular fibrillation	- CIED dose 2–10 Gy without pacemaker dependency or frequent ICD intervention - CIED dose ≤ 10 Gy without pacemaker dependency but with frequent ICD intervention or RT with protons or photons > 6 MV - CIED dose ≤ 2 Gy with pacemaker dependency	- CIED dose ≤ 5 Gy with pacemaker dependency and/or production of secondary neutrons	- ICD dose < 5 Gy - PM dose < 5 Gy with pacemaker dependency	- CIED dose ≤ 5 Gy with pacemaker dependency	- CIED dose 2– 5 Gy without pacemaker dependency - CIED dose ≤ 5 Gy with pacemaker dependency
	High risk	- CIED dose > 10 Gy - CIED dose > 2 Gy with pacemaker dependency or history of prior ventricular fibrillation	- CIED dose > 10 Gy - CIED dose > 2 Gy with pacemaker dependency or history of prior ventricular fibrillation	- CIED dose > 10 Gy - CIED dose > 2 Gy with pacemaker dependency - CIED any dose with pacemaker dependency/frequent ICD intervention and RT with protons or photons > 6 MV	- CIED dose > 5 Gy	- CIED dose ≥ 5 Gy - RT with ≥10 MV	- CIED dose > 5 Gy - RT with production of secondary neutrons	- CIED dose > 5 Gy - RT with production of secondary neutrons
PM-dependency as independent risk factor		Yes	Yes	Yes	Yes	Yes	Yes	Yes
Secondary neutron production as independent risk factor		No *	No *	Yes	Yes	Yes	Yes	Yes
Emergency protocol required		Yes	Yes ^†^	Yes	No	No	No	Yes
Frequency of interrogations, monitoring and precaution stratified by risk group								
	Low risk	- interrogation after every RT session - personnel qualified for specific procedures in respect to CIED patients	- weekly interrogations - audiovisual contact - deactivation of ATP therapy of ICDs through reprogramming or magnet placement during RT	- first and mid-treatment interrogations - audiovisual contact	- interrogation only at the end of RT	- interrogations every 2 weeks - disabling (temporarily) of the “R” function, automatic measurement and setting safe stimulation impulses (with a margin of at least 1 V above the stimulation threshold)	- interrogation after the last RT session	- interrogation before first and after last RT session
	Intermediate risk	- interrogation before and after every RT session - ECG and SpO2 monitoring - external defibrillator and external pacemaker available, programming device available	- weekly interrogations - medical emergency cart available - external pacemaker available - trained staff with cardiology expertise can be present within 10 min	- first and mid-treatment interrogations - ECG and SpO2 monitoring	- weekly interrogations	- weekly interrogations - setting stimulation to a frequency other than the default frequency of a reset device - presence of a cardiologist experienced in the use of CIED during the first RT session	- consider weekly interrogations	- additional interrogation at mid-treatment - formal consultation with cardiology/electrophysiology - pacing-dependent: consultation with cardiology/electrophysiology on the use of magnet and SpO2
	High risk	- interrogation before and immediately after every RT session - device relocation or replanning of RT with dose reduction at CIED - if reduction of CIED dose is impossible then consider RT on individual basis - cardiologist or anesthesiologist present	- in exceptional cases a decision to start RT can be made - interrogations within 24 h after every RT session - ECG-monitoring during RT	- weekly interrogations in addition to the first and mid-treatment interrogations	- weekly interrogations - telemetry monitoring - presence of cardiologist/intensivist - magnet placement depending at the discretion of the cardiologist	- interrogation immediately before and after completing every RT session - presence of cardiologist during RT	- weekly interrogations	- weekly interrogations once the device receives >5 Gy - weekly ECG monitoring - cardiologist/pacemaker technologist should be available, if needed
Interrogations, monitoring and precaution independent of risk group								
	During RT	- RT with photon beam energy ≤ 10 MV - evaluation of RT dose at CIED during first RT session and comparison with calculated CIED dose - deactivation of ATP therapy of ICDs through reprogramming or magnet placement during RT - audiovisual contact - continuous ECG and SpO2 monitoring in patients with suspended ATP therapy - availability of cardiologist and programming device	- RT with photon beam energy ≤ 10 MV	- interrogation in office/remote after the first RT session - interrogation at mid-treatment	- medical emergency cart available with physician on-site - audiovisual contact during RT - telemetric surveillance if available	- RT with photon beam energy < 10 MV - access to external defibrillator with external stimulation option - audiovisual contact, ECG, BP and SpO2 monitoring in high-risk patients, readiness for resuscitation - maintaining contact with the programmer - deactivation of ATP therapy of ICDs through reprogramming or magnet placement during RT - evaluation of the RT dose during the first RT sessions	- audiovisual contact - CIED relocation if it interferes with adequate tumor treatment - CIED relocation not recommended for CIED with dose <5 Gy - non–neutron-producing treatment preferred over neutron-producing treatment	- PM magnet, SpO2 and AED available - audiovisual contact - communication with cardiology/electrophysiology
	After RT	- interrogation of CIED after the last RT session - checkups of CIED at 1, 3 and 6 months after RT - asynchronous stimulation not longer than necessary - analysis of any CIED irregularities in connection to RT and forwarding of data to manufacturer - exchange of CIEDs with significant defects even if the malfunction is temporary and full device recovery is observed - telemetric surveillance if available	- interrogation of CIED after the last RT session - checkups of CIED at 1, 3 and 6 months after RT	- interrogation of CIED after the last RT session - checkups of CIED at 1 and 6 months after RT	- interrogation of CIED after the last RT session - checkup of CIED 3–6 months after RT	- interrogation of CIED after the last RT session - checkups of CIED at 1, 3 and 6 months after RT	- interrogation of CIED after the last RT session	- interrogation of CIED after the last RT session - checkups of CIED at 1 and 6 months after RT
	To be additionally considered	- asynchronous mode in stimulation-dependent patients	- device relocation - measurement of RT dose at CIED site	- magnet placement - device reprogramming - device relocation - presence of electrophysiologist/nurse/technician - presence of anesthesiologist	- magnet placement only at the discretion of the cardiologist	- asynchronous mode in stimulation-dependent patients - considering device replacement in case of damage		- deactivation of ATP therapy of ICDs through reprogramming or magnet placement during RT at the discretion of the cardiologist

Only the most important recommendations are summarized here. * Only radiotherapy without production of secondary neutrons should be performed according to the guideline. † Resuscitation protocols are required. Abbreviations: AAPM = American Association of Physicists in Medicine; AIAC = Italian Association of Arrhythmology and Cardiostimulation; AIFM = Italian Association of Medical Physics; AIRO = Italian Association of Radiation Oncology; ATP = antitachycardia pacing; BP = blood pressure; CIED = cardiac implantable electronic device; DEGRO = German Society for Radiation Oncology; DKG = German Cardiac Society; ECG = electrocardiogram; HRS = Heart Rhythm Society; PM = pacemaker; PTK = Polish Cardiac Society; PTRO = Polish Society of Radiation Oncology; RT = radiotherapy; SFRO = French Society of Radiation Oncologists; SpO2 = pulsoximetry.

## Data Availability

The research data are stored in an institutional repository and will be shared upon request to the corresponding author.

## Supplementary Materials

The following supporting information can be downloaded at: https://www.mdpi.com/article/10.3390/cancers15194830/s1, Table S1: Patient characteristics. Abbreviations: CIED = cardiac implantable electronic device; CRT = cardiac resynchronization therapy; DCM = dilated cardiomyopathy; HF = heart failure; ICD = implantable cardioverter defibrillator; ICM = ischemic cardiomyopathy; IQR = interquartile range; KPS = Karnofsky performance status; PM = pacemaker; SSS = sick sinus syndrome; TBS = tachycardia-bradycardia syndrome; AAI(R)/DDD(R)/DDI(R)/VDD(R)/VVI(R) = Different modes of cardiac pacing.
